# Intussusception due to non Hodgkin’s lymphoma; different experiences in two children: two case reports

**DOI:** 10.4076/1757-1626-2-6304

**Published:** 2009-09-01

**Authors:** Vikal Chandra Shakya, Chandra Shekhar Agrawal, Rabin Koirala, Sudeep Khaniya, Saroj Rajbanshi, Sagar Raj Pandey, Shailesh Adhikary

**Affiliations:** 1 Department of Surgery, B P Koirala Institute of Health SciencesDharanNepal; 2 Department of Pathology, B P Koirala Institute of Health SciencesDharanNepal

## Abstract

In children, non-Hodgkin’s lymphoma has been found to be the lead point in intussusception involving the terminal ileum. We present here two cases of ileal primary non-Hodgkin’s lymphoma which presented as intussusception, highlighting the differing presentations of these children and their outcome, with a brief review of the literature.

## Introduction

Intussusception is a pathology in which telescoping of a proximal segment of bowel occurs into lumen of the distal segment. The incidence of intussusception is 1.5-4 cases per 1000 live births, with a male-to-female ratio of 3:2 [1,2]. It often occurs around 1 year of age, with a peak incidence between 4 and 7 months [3]. In infants aged 9-24 months, it is usually primary i.e. they do not have an identifiable specific lead point. A specific lead point is more commonly found in children older than 3 years [4]. Though uncommon, primary Non Hodgkin’s lymphoma (NHL) is found to be the lead point in intussusception, commonly involving the terminal ileum [5,6]. Here we present two cases of ileal primary Non Hodgkin’s lymphoma which presented as intussusception, and we would like to share their presentations and outcome, and our experience in the management of these children, with a brief review of the literature.

## Case presentation

### Case report 1

A 3-year-old male Nepalese child of Mongolian ethnicity reported to the emergency room with complaint of crampy abdominal pain in lower right quadrant and umbilical region progressively increasing in intensity for last 3 days, nausea, vomiting and passage of loose stool with blood. On clinical examination, abdomen was distended and tender with signs of peritoneal reaction. Bowel sounds were exaggerated. Patient’s rectal examination revealed red currant jelly, and ballooning of rectum. Laboratory investigations showed total leucocyte counts of 14,000/mm^3^ with neutrophilla. Chest X-ray was normal; abdominal plain radiograph showed dilated bowel loops with multiple air fluid levels. USG abdomen revealed bowel intussusception at the right lumbar region, extending to the epigastrium, along with mesenteric lymphadenopathy.

**Figure 1.**
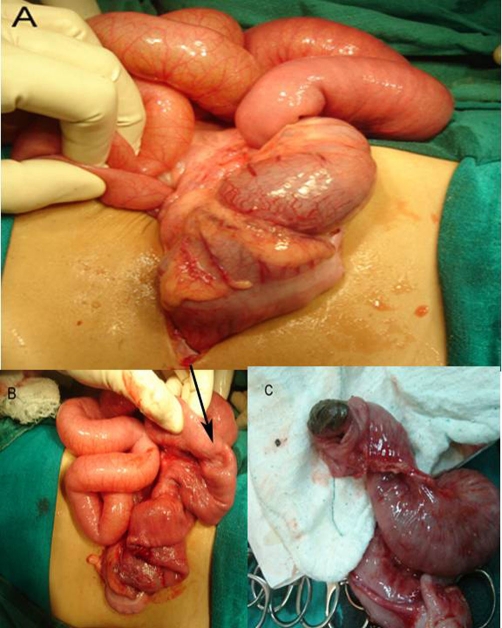
**(A)** Photograph showing the ileoileocolic intussusception, **(B)** the thickened area as seen from the outside, and **(C)** the cut open section showing the sessile polyp.

**Figure 2.**
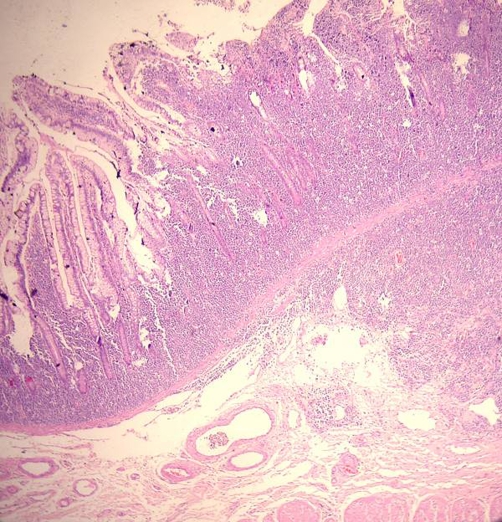
Photomicrograph showing B-cell lymphoma cells in the intestinal mucosa & submucosa (×200 H & E stain).

**Figure 3.**
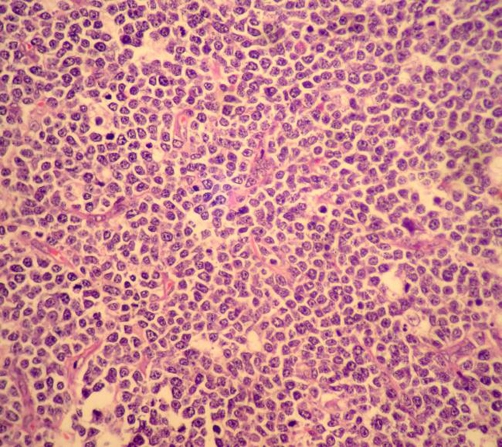
Photomicrograph showing B-cell lymphoma cells in the intestinal mucosa (×400 H & E stain).

**Figure 4.**
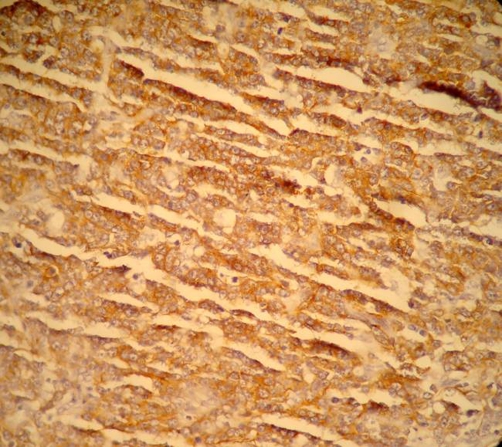
Photomicrograph showing cells positive for B-cell marker (CD20) (×400).

The child was taken to theatre for emergency exploration which confirmed ileoileocolic intussusception with a small (1.5 cm × 1.5 cm) submucosal sessile polyp, firm in consistency, in the distal ileum as the leading point along with mild congestion and hemorrhage in the mucosa (Figure 1). Mesenteric lymph nodes were enlarged. Right hemicolectomy was done and the child made rapid uneventful recovery. Histopathological examination of the polypoid lesion showed features of diffuse large B-cell Non Hodgkin lymphoma (Figures 2,3,4). Rest of the bowel was normal. Isolated mesenteric nodes were reactive. The total leucocyte count came back to normal later. The peripheral cytology smear and bone marrow aspiration were also normal. CT scan of the chest showed no mediastinal lymphadenopathy. The disease was staged as primary ileal Stage 1 disease according to Ann-Arbor classification and he was put on post operative chemotherapy. The child is on regular follow up for last 12 months and has not developed any symptoms.

### Case report 2

A 2-year-old male Nepalese child of Aryan ethnicity was brought to the emergency room with complaints of whole abdominal pain, passage of red currant jelly and vomiting of 4 days duration. The child was pale, dehydrated, there was no peripheral lymphadenopathy, the abdomen was distended and tender with signs of peritoneal reaction, and the bowel sounds were absent. Rectal examination revealed red currant jelly with ballooning of rectum. Laboratory investigations showed hemoglobin of 4 gm% and TLC 25,000/mm^3^ with neutrophilla. Chest X-ray was normal; abdominal plain films showed features of small bowel obstruction. USG abdomen showed target sign at the right lumbar region extending to the epigastrium, suggestive of intussusception alongwith enlarged mesenteric lymph nodes.

**Figure 5.**
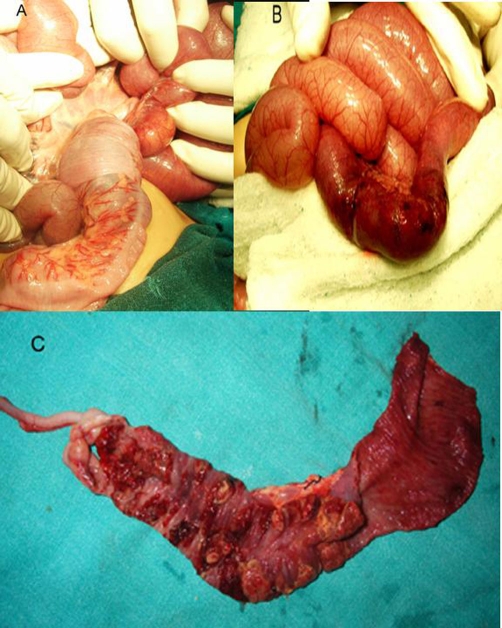
**(A)** Photograph showing the ileocolic intussusception, **(B)** congested intestine and patchy gangrenous changes, and **(C)** the cut open section showing multiple localized nodules in the bowel wall, with patches of intestinal necrosis.

After resuscitation, the child was taken to theatre for emergency exploration which confirmed ileocolic intussusception with congested mucosa and patchy gangrenous changes (Figure 5). There were multiple localized nodular thickenings of the bowel wall, with patches of intestinal necrosis and mesenteric lymphadenopathy. Right hemicolectomy was done. Postoperatively, the total leucocyte counts continued to be high, the peripheral blood smear no other features than leukocytosis, he had persistent abdominal distension, and he was found to develop anastomotic leak on the 6th postoperative day. He expired on the 8th postoperative day due to sepsis. Histopathological examination of the nodules in the resected bowel revealed features of diffuse large B-cell Non Hodgkin’s lymphoma along with congested, edematous mucosa and gangrenous changes. The mesenteric nodes were reactive. Due to early death of the patient, staging could not be properly done, but the child was considered to be suffering from primary ileal Non Hodgkin’s lymphoma, which was locally disseminated, supported by absence of peripheral lymphadenopathy, normal peripheral blood picture, and normal chest radiograph.

## Discussion

Primary Non Hodgkin’s Lymphoma of the gastrointestinal tract is the most common extranodal lymphoma [7]. Small and large intestines are the most frequent sites of involvement in the pediatric age group [8]. It is most commonly found in the ileum, where the greatest concentration of gut-associated lymphoid tissue is present. They are commonly derived from B-cells from the lymphoid tissue present in the lamina propria and submucosa. They may be solitary or diffuse; solitary form tends to encircle the bowel and narrow the lumen, diffuse form shows multi-segment involvement with numerous polypoidal excersences. It may then invade the serosa to mesentery or beyond.

The most common lead point in intussusception has been found to be the Meckel’s diverticulum [4-6,9-11]. Other lead points that have been reported are polyps, duplication cyst, carcinoid, leiomyoma, hemangioma, fibrosarcoma and buried appendectomy stump [5,6,10]. It can also occur in association with a diffuse process including Henoch-Schonlein purpura, celiac disease, neutropenic colitis, cystic fibrosis, and Peutz-Jehgers syndrome [12-14]. The incidence of NHL acting as a lead point in intussusception is reported to be as high as 17%, and even higher (more than 50%) in children over 4-6 yrs of age [4,9,11].

In the western population, 60% to 80% of intestinal lymphomas have been found to be B-cell lymphomas, mostly diffuse large B-cell lymphoma of the distal small intestine [15,16]. In our study also both patients had diffuse large B-cell lymphoma. In the first case, there was a sessile polyp in the distal ileum, the child went home after a right hemicolectomy, and is well after chemotherapy. However in the second case, there was multiple segmental involvement with gangrenous bowel, resection and anastomosis later resulted in leak and sepsis in the postoperative period. The bowel involvement was more extensive in the second case; this was probably the decisive factor in the outcome of the child. With the combination of NHL with intussusception, previous author’s experiences have not been very satisfactory either. In Ein SH et al’s study, only 3 out of 10 children were long term survivors [11]. Puri P et al also reported that the only one death out of entire series of 292 children with intussusception was a child with lymphoma [6]. Lymphoma is the most common malignant lesion of small bowel in children [6]. Hence, in cases of intussusception, especially in the older age group of the children, we need to keep a high index of suspicion for malignant lymphoma of the bowel. The importance of resection of bowel containing any slightest lesion, along with removal of the regional lymph nodes, is stressed. With these case reports we have tried to highlight the differing presentations of NHL and the differing unpredictable outcomes. In fact, LaQuaglia et al in his study has concluded that bowel resection performed during emergency laparotomies for symptomatic, localized bowel involvement in patients with NHL was associated with better prognosis [15]. Intestinal involvement by NHL was associated with an increased frequency of abdominal symptoms resulting in earlier laparotomies and earlier diagnosis. A mesenteric or retroperitoneal mass that does not involve the bowel wall remains clinically silent until a relatively larger tumor burden is reached. Complete resection of the tumor was shown to have the added advantage of avoiding bowel perforation, gastrointestinal hemorrhage or the tumor-lysis syndrome after the initiation of chemotherapy [17-19]. In NHL involving the bowel, surgical resection has been associated with improved outlook by complete resection in localized disease confined to the bowel wall and diagnostic biopsy in advanced diseases [20]. Hence, with high suspicion of lesions, considering the age of the child and the bowel involvement, resection of the diseased bowel may be the single most important decision in salvaging these children. Role of debulking has been condemned in case of extensive abdominal tumors, which is associated with a greater complication rate and possible delay in initiation of essential systemic chemotherapy [15]. Extent of disease at presentation and the resectability has been found to be the most important prognostic factor [15,20].

## Abbreviations

NHL: non-Hodgkin’s lymphoma
TLC: total Leucocyte count
USG: ultrasonography
